# Diagnosing an atypical case of tuberculosis presenting as dacryocystitis: Sac biopsy and GeneXpert

**DOI:** 10.22336/rjo.2022.63

**Published:** 2022

**Authors:** Gautam Lokdarshi, Seema Kashyap, Nripen Gaur

**Affiliations:** * Oculoplastic and Ocular Oncology Services, IRIS Superspeciality Eye Hospital, Line Tank Road, Ranchi, India; ** Ocular Pathology Services, Dr. R. P. Centre for Ophthalmic Sciences, All India Institute of Medical Sciences, Ansari Nagar, New Delhi, India; *** Oculoplasty & Pediatric Ophthalmology Services, Dr. R. P. Centre for Ophthalmic Sciences, All India Institute of Medical Sciences, Ansari Nagar, New Delhi, India

**Keywords:** tuberculous dacryocystitis, Sac biopsy, GeneXpert, CT-DCG, granulomatous dacryocystitis

## Abstract

**Objective:** To present an atypical case of tuberculous dacryocystitis.

**Method:** An adult female presented with long standing epiphora with gradual swelling over lacrimal sac region. On syringing, water was felt in throat with no regurgitation. CT-DCG and CECT orbit were done subsequently and simultaneously. Ill-defined, enhancing soft tissue surrounding and involving the lacrimal sac wall was identified. The sac wall outline was seen distorted with contrast in NLD. The histopathology was suggestive of non-specific chronic inflammation. GeneXpert analysis was shown to be very low positive for M. tuberculosis. Montoux test was strongly positive (40 x 40 mm). ATT was started.

**Results:** The swelling and watering subsided over the next few months.

**Conclusion:** Tuberculosis should be considered in cases of chronic granulomatous dacryocystitis. CECT with CT-DCG is essential imaging. GeneXpert is a new and sensitive tool with considerable specificity in cases in which histopathology is not conclusive. ATT is curative and DCR is reserved for only unresolved NLDO with persistent epiphora.

## Introduction

Diagnosing tuberculosis of lacrimal sac can be cumbersome in patients with epiphora. A high degree of clinical suspicion should be kept for patients from endemic regions. Herein, we present a case of epiphora, in which the detailed clinical workup revealed the sac tuberculosis and saved the patient from unwanted (dacryocystorhinostomy) surgery.

## Case report

**Fig. 1:**
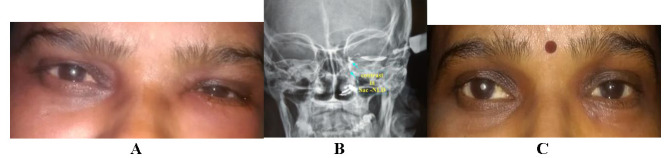
**A-C** A non-inflamed swelling over left lacrimal sac area; **B.** The swelling disappeared completely after four months after starting ATT; **C.** Contrast seen in NLD and inferior meatus on X-rays DCG

A female in her 40’s came to our outpatient department for a second opinion on her left side dacryocystorhinostomy (DCR) surgery that was advised elsewhere. She presented with left eye epiphora that she had for the last six months. This was associated with painless, gradual swelling over the left medial canthus region. On inspection it appeared as a fullness in the lacrimal sac area, which was firm and non-tender on palpation (**Fig. 1 A**. No redness, warmth, discharge or regurgitation of sac on pressure was noted. Both puncta appeared normal on slit lamp examination. On syringing, water was felt in the throat with no regurgitation or delay. No regional lymph node enlargement, history of fever, immunosuppression or systemic illness were noted.

**Fig. 2:**
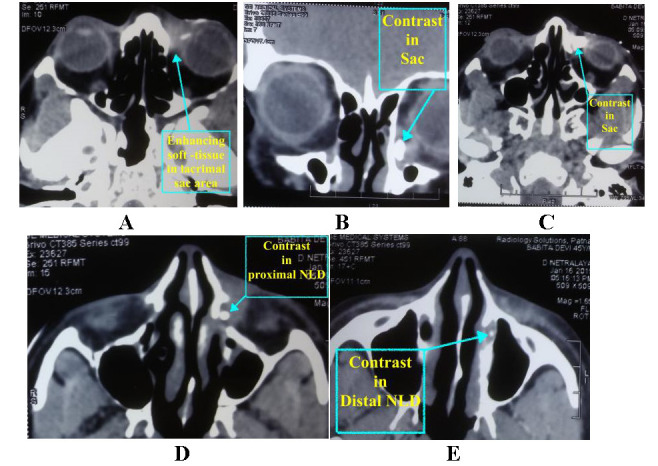
**A-E** Ill-defined soft tissue enhancement in the left lacrimal sac region on CECT **(A)**; Contrast seen in the left lacrimal sac **(B, C)**, proximal **(D)**, and distal NLD **(E)** on CT-DCG

X-rays Dacryocystography (DCG) revealed patent but mildly irregular sac lumen (**Fig. 1 B**). CT-DCG and CECT orbit were done subsequently and simultaneously. On CECT, ill-defined, enhancing soft tissue surrounding and involving left canaliculi and the lacrimal sac wall, was identified (**Fig. 2 A**). The sac wall outline was seen distorted with similar soft tissue enhancement seen inside the nasolacrimal duct (NLD). Minimal post-septal extension (i.e. behind posterior lacrimal crest) was noted. There was no frank necrotic area or bony change. Paranasal Sinuses appeared normal. On CT-DCG, contrast was seen inside the left sac, NLD and inferior meatus (**Fig. 2 B-E**).

Incisional biopsy was performed through standard dacryocystorhinostomy (DCR) incision. The biopsy specimen was taken from the sac wall in full thickness. Sero-mucoid discharge samples from the inside of the sac were collected. Hematoxylin-eosin (H&E) stain, Periodic acid Schiff (PAS) stain and Cartridge-based nucleic acid amplification test (CBNAAT, also k.a. GeneXpert) were performed on the biopsy specimen. Gram stain, KOH mount, Zeil-Neilson (Z-N) Stain, Bacterial culture (Blood agar and Chocolate agar) and Fungal Culture (SDA) were performed on both the biopsy specimen and the discharge samples.

## Results

**Fig. 3:**
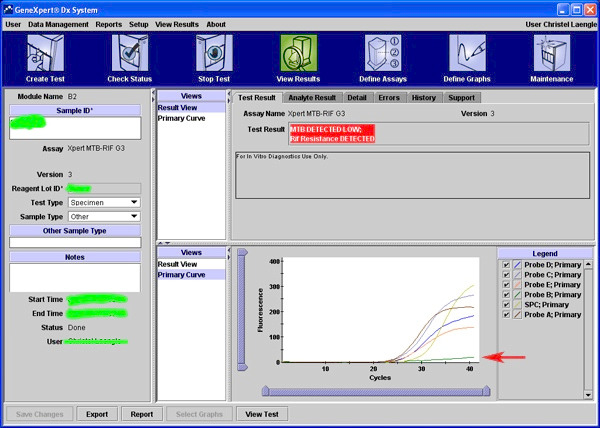
Screenshot of GeneXpert result for the sample showing “MTB DETECTED LOW, Ref. Resistance NOT detected” (red arrow points lower titer)

The histopathology was suggestive of non-specific chronic inflammation. Gene Xpert analysis was shown to be very low positive for *M. tuberculosis* (**Fig. 3**). No resistance to Rifampicin was found. Montoux test was strongly positive (40 x 40 mm induration with redness at 72 hours). Stains and cultures results were inconclusive. Pulmonary physician and Otolaryngologist consultations were done to rule out any other/ systemic foci. Category I Antitubercular treatment (ATT) i.e. 2 HRZE + 4 HR was started for six months. Improvement was noticed in the first month with complete resolution of all symptoms within the first four months (**Fig. 1 C**). Visual acuity, colour vision, contrast sensitivity and fundoscopy were normal on monthly follow-ups. No investigation was performed at completion of ATT course. No recurrence was reported after one-year drug-free period on three monthly follow-ups.

## Discussion

Sac tuberculosis has been underreported in India, where tuberculosis is endemic. Most of the reported cases were suspected by surgeons while performing dacryocystorhinostomy and were diagnosed post-operatively [**1**]. This could be because of a relatively high prevalence of primary acquired nasolacrimal duct obstruction (PANDO), non-specific long-standing symptoms, sub-clinical infection, and unproper evaluation with adequate pre-operative clinical or radiological studies especially in failed DCR cases [**2**,**3**]. In our case, the absence of redness, warmth, pain and tenderness ruled out acute inflammation. The firm nature in the absence of discharge or regurgitation ruled out the possibility of dacryocystitis or mucocele. As a standard practice, syringing must be done to rule out mechanical blockade before taking the decision for surgery, which was not mentioned in some previous reported new cases [**3**,**4**].

CECT and CT-DCG in same sitting help in the further planning in the atypical presentation of epiphora [**3**]. X-rays DCG, less informative than CT-scan, can be done (optional), for advantages like less radiation, being cheap and fast, or when the CT facility is not available.

Microbiology and pathology may not be conclusive in tuberculous lesion, which could be due to low bacterial load, fastidious nature of the bacteria, inadequate sample, etc. GeneXpert (CBNAAT), a real time polymerase chain reaction (PCR), is a rapidly feasible test, highly sensitive and specific in detecting traces of *mycobacterium* especially in smear negative tuberculosis (TB). WHO recommended the test for TB, which can detect rifampicin resistance as well [**5**].

Strongly positive Montoux test (> 15 mm) alone, without identifiable risk factors, is suggestive of active TB. Sometimes, lupus vulgaris overlying sac lesion could raise suspicion of active tuberculosis of the sac and may have a strongly positive Montoux test [**6**].

It is not necessary to have systemic foci, isolated or primary sac tuberculosis (TB) has been reported, but should be ruled out by pulmonary physician and otolaryngologist [**4**]. Nasal mucosa involvement has been reported in several published cases of sac tuberculosis, many among them being documented after primary DCR surgery [**2**]. So, whether it was “primary” nasal mucosal lesion or “secondary” lesion spread from sac directly, is not clear yet.

As for standard teaching, opening the sac before making the bony ostium, inspecting its contents and inner walls/ sac-flap in full length under operating microscope during external DCR, is essential [**7**,**8**]. If any suspicious tissue suggesting granuloma, caseous material, bone erosion or mass are found, full-thickness incisional biopsy from the sac wall must be sent for histopathology and microbiology, without proceeding to osteotomy [**2**,**7**]. If the laboratory reports are favorable, then only one can safely proceed to DCR. We believe that osteotomy breaches compartmentalization of the lesion and mucosal anastomosis provides free passage for direct spread of tuberculous granuloma to nasal cavity. 

Anti-tubercular regime is curative and should be followed as per WHO guidelines. Surgery, either dacryocystorhinostomy or dacryocystectomy, is not indicated primarily and should be reserved for persistent epiphora after completion of ATT course, especially for the epicenter in nasolacrimal duct or inferior turbinate [**2**,**9**].

## Conclusion

To conclude, tuberculosis should be considered in cases of chronic granulomatous dacryocystitis. CECT with CT-DCG is essential imaging. GeneXpert is a new and sensitive tool with considerable specificity in such cases in which histopathology is not conclusive.

**Conflict of Interest statement**

The authors state no conflict of interest.

**Informed Consent and Human and Animal Rights statement**

An informed consent was taken from the patient for publishing the clinical photographs.

**Authorization for the use of human subjects**

Ethical approval: The research related to human use complies with all the relevant national regulations, institutional policies, is in accordance with the tenets of the Helsinki Declaration, and has been approved by the review board of Oculoplastic and Ocular Oncology Services, IRIS Superspeciality Eye Hospital, Ranchi, India.

**Acknowledgements**

None.

**Sources of Funding**

None.

**Disclosures**

None.
